# Microvascular function in pre‐eclampsia is influenced by insulin resistance and an imbalance of angiogenic mediators

**DOI:** 10.14814/phy2.13185

**Published:** 2017-04-28

**Authors:** Anshuman Ghosh, Nicholas S. Freestone, Nicholas Anim‐Nyame, Francesca I. F. Arrigoni

**Affiliations:** ^1^ School of Life SciencesPharmacy and ChemistryKingston University LondonKingston upon ThamesUK; ^2^ Department of Obstetrics & GynaecologyKingston HospitalKingston upon ThamesUK

**Keywords:** Angiogenesis, insulin resistance, microvascular, pregnancy/preeclampsia

## Abstract

In preeclampsia, maternal microvascular function is disrupted and angiogenesis is dysfunctional. Insulin resistance that occurs in some pregnancies also pathologically affects microvascular function. We wished to examine the relationship of angiogenic mediators and insulin resistance on microvascular health in pregnancy. We performed a nested, case–control study of 16 women who developed preeclampsia with 17 normal pregnant controls. We hypothesized that the impaired microvascular blood flow in preeclamptic women associated with an increased ratio of the antiangiogenic factors; (s‐endoglin [sEng] and soluble fms‐like tyrosine kinase‐1 [sFlt‐1]) and proangiogenic molecule (placental growth factor [PlGF]) could be influenced by insulin resistance. Serum samples taken after 28 weeks of gestation were measured for the angiogenic factors, insulin, and glucose alongside the inflammatory marker; tumor necrosis factor‐α and endothelial activation, namely; soluble vascular cell adhesion molecule 1, intercellular adhesion molecule‐1, and e‐selectin. Maternal microvascular blood flow, measured by strain gauge plethysmography, correlated with ratios of pro‐ and antiangiogenic mediators independently of preeclampsia. Decreased microvascular function measured in preeclampsia strongly correlated with both the antiangiogenic factor (sFlt‐1 + sEng): PlGF ratio and high levels of insulin resistance, and combining insulin resistance with antiangiogenic factor ratios further strengthened this relationship. In pregnancy, microvascular blood flow is strongly associated with perturbations in pro‐ and antiangiogenic mediators. In preeclampsia, the relationship of maternal microvascular dysfunction with antiangiogenic mediators is strengthened when combined with insulin resistance.

## Introduction

In preeclampsia, generalized maternal endothelial dysfunction (Roberts et al. 1991) and inflammation is widespread (Chambers et al. 2001) resulting in circulatory changes (Roberts and Redman 1993; Levine et al. 2004) influenced by factor(s) released by the ischemic placenta (Anim‐Nyame et al. 2015a, 2015b). The resulting multisystem presentation of end organ damage, suggestive of an underlying microvascular dysfunction, previously demonstrated by our group (Anim‐Nyame et al. 2003) is preceded by reduced tissue perfusion (Anim‐Nyame et al. 2001). Maternal endothelial dysfunction is considered to be influenced either by the effects of mediators on a preexisting susceptible vasculature prior to the manifestation of clinical preeclampsia (Savvidou et al. 2003) or by their direct impact on the vasculature during pregnancy.

An elevation of circulating antiangiogenic factors concomitantly with a decrease in proangiogenic factors has been found in preeclampsia and is associated with vascular endothelial dysfunction; however, how this affects the microvasculature is unclear.

In addition to the influence of circulating angiogenic factors on microvascular function in preeclampsia, epidemiological studies have demonstrated an increased risk of preeclampsia in pregnancies complicated by insulin resistance (Caruso et al. 1999; Wolf et al. 2002; Seely and Solomon 2003). Gestational insulin resistance associated with preeclampsia has been associated with vascular dysfunction in large and small vessels (Anim‐Nyame et al. 2015b). How insulin resistance may contribute to preeclampsia is not well defined but there is a suggestion that angiogenic and insulin‐dependent pathways may influence each other (Gerber et al. 1998; Kandel and Hay 1999; Autiero et al. 2003) with angiogenesis and insulin resistance sharing molecular mechanisms of action (Chou et al. 2002; Montagnani et al. 2002; Vicent et al. 2003). Moreover, the proangiogenic effect of PlGF is strongly influenced and modified by insulin resistance (Thadhani et al. 2004).

In an effort to identify biomarkers that are pathologically linked to preeclampsia and associated vascular function, we tested the hypothesis that an interaction exists between microvascular function, angiogenesis, and insulin resistance in normal and preeclamptic pregnancies in a nested case–control study.

## Materials and Methods

In this prospective study of 33 pregnant women, 16 preeclamptic and 17 normotensive participants were recruited after 28 weeks of gestation. The women were similar in maternal age, gestational age, booking body mass index (BMI), and ethnicity as previously described (Anim‐Nyame et al. 2015a).

Preeclampsia was defined as new onset hypertension and proteinuria.

### Participants

This prospective study enrolled women after 28 weeks of gestation. Hypertension was defined as systolic blood pressure ≥140 mmHg or diastolic blood pressure ≥90 mmHg, measured on at least two occasions, at least 4 h apart. Significant proteinuria was defined as urinary protein excretion >300 mg/24 h (Chaiworapongsa et al. 2013).

Power calculations were based on a previous cross‐sectional study, with resting blood flow significantly reduced in preeclampsia compared to normal pregnant controls (1.95 ± 0.9 mL/min/100 mL vs. 3.9 ± 1.4 mL/min/100 mL, *P* = 0.004, for preeclampsia and normal pregnancy, respectively) (Anim‐Nyame et al. 2000). Thus, a sample size of 10 in each group was sufficient to achieve statistical significance with *α* of 0.05 and *β* of 0.02. The South London and Borders Local Ethics Committee approved the study, and informed consent was obtained from each participant. The study conformed to the Helsinki Declaration.

Women with gestational diabetes or other metabolic, cardiovascular, inflammatory, immune, infectious, or neoplastic conditions were excluded from the study, as were smokers. The obstetric records of the women were reviewed after delivery to confirm reversal of hypertension and proteinuria in the preeclamptic group.

### Blood sampling and assays

Blood samples were obtained from the antecubital vein of each participant using aseptic techniques and collected into EDTA tubes (4 mL) (Becton Dickenson, Vacutainer System, UK). The serum was separated by centrifugation at 1500 *g* for 10 min and stored at −80°C until assayed.

Human PlGF, sEng, sFlt‐1, sICAM, sVCAM, e‐selectin, and TNF‐*α* were measured by an enzyme linked immunosorbent assay (ELISA) (R&D System Europe, U.K.). These assays detect‐free, but not bound growth factors. All samples were collected, handled, and stored under the same conditions and tested in duplicate. The wells were read within 30 min using a plate reader at a wavelength of 450 nm as per the manufacturer's guidelines (Labtech International, East Sussex, UK).

Microvascular blood flow was measured by strain gauge plethysmography and determination of insulin resistance was calculated from fasting maternal plasma glucose and insulin concentrations. Insulin resistance was calculated using the homeostasis model assessment (HOMA).

### Measurement of blood flow

The study was performed in a quiet room with the temperature kept constant between 24 and 25°C. Participants were rested for at least 15 min before the study and blood pressure was measured in triplicate with the mean reading being used (Dinamap Vital Sign Monitor, Critikon, Fl). Observations were made in the left lateral position to prevent aortocaval compression, and with the right midcalf supported at the level of the heart. Blood flow was measured using a Filtrass strain gauge plethysmograph (Filtrass; DOMED, Munich, Germany) (Anim‐Nyame et al. 2001). The device is mercury‐free, with an integrated automatic calibration function that allows a touch‐free calibration, thus reducing artifacts due to investigator manipulation. The sensor was calibrated automatically in triplicate by a computer‐driven program at the start of each study. The Filtrass program is computer‐assisted and allows the selection of prerecorded protocols for measuring blood flow in the calf. The congestion pressure cuff, which is attached to a built‐in compressor pump, was placed around the right thigh and enclosed in a rigid corset to reduce filling volume and thus filling time. Changes in calf circumference in response to a rapid increase in thigh cuff pressure were measured using a passive inductive transducer with an accuracy of ±5 *μ*m. The files were saved for subsequent off‐line analysis.

Calf blood flow, measured in mL·min^−1^·100 mL^−1^, was measured using a protocol that has been described previously (Anim‐Nyame et al. 2001). The venous congestion pressure was raised rapidly to 40 mmHg and the pressure held for 20 sec. Since this pressure occludes venous return but not arterial blood inflow, the initial swelling rate equals arterial blood flow. The change in circumference of the calf was estimated from the slope of the first 3 sec of the volume response to the pressure step. The flow was measured at 3 sec ‐cuff inflation to remove variabilities in the measurement of blood flow without the influence of changes in capillary filtration and venous compliance (Skoog et al. 2015).

This procedure was repeated three times with the congestion pressure kept at zero for 5 min between each measurement. The system's analysis program calculates the change in circumference and uses it to estimate volume change, assuming that the segment of calf being studied is a cylinder of uniform diameter and constant length. An ankle occlusion cuff was not used to exclude contributions from non‐nutritive flow through arteriovenous shunts of the feet. Maternal tissue blood flow was measured in the calf, as it has a high muscle to skin ratio, meaning blood flow through it relates to metabolic requirements rather than thermoregulation. Moreover, the skeletal muscle lacks arteriovenous channels and therefore most of the measured arterial flow will traverse the microvascular bed, thus representing the microvascular blood flow.

### Statistical analysis

Normally distributed data were expressed as mean ± SEM and nonparametric data as median and interquartile ranges. Univariate comparisons between groups were analyzed using the Mann–Whitney test. For univariate comparisons of blood flow analysis with immunoassay data, the Kruskall–Wallis test was used.

Bivariate correlations were determined using Pearson or Spearman correlation coefficients as appropriate. Multivariable logistic regression analyses explored relationships between microvascular blood flow and circulating mediators. Effect modification was examined using interaction terms in the logistic regression model. Values of *P* < 0.05 were considered statistically significant. Statistical analysis was performed using SPSS version 22 (SPSS Inc., Chicago, Ill).

## Results

There was no significant difference in maternal age, booking body mass index (BMI), or hematocrit between the two groups (Table 1). As expected from the recruitment criteria, women with preeclampsia had higher systolic blood pressures (140.6 ± 2.06 mmHg vs. 112.8 ± 2.87 mmHg, *P *<* *0.0001; preeclamptic and normotensive women, respectively) and diastolic blood pressures (94.6 ± 1.16 vs. 75.5 ± 1.88 mmHg, *P *<* *0.0001; preeclamptic and normotensive women, respectively) when compared with normotensive pregnant women.

**Table 1:** Demographic and clinical data

Variable	Normal pregnancy (*n* = 17)	Preeclampsia (*n* = 16)	*P* value*
Age (years)	33.24 ± 1.28	34.11 ± 0.52	NS
BMI (kg/m^2^)	25.87 ± 0.95	23.87 ± 0.91	NS
Systolic BP (mmHg)	112.76 ± 2.87	140.63 ± 2.06	<0.0001*
Diastolic BP (mmHg)	75.47 ± 1.88	94.56 ± 1.16	<0.0001*
Mean arterial pressure (mmHg)	87.90 ± 1.92	109.92 ± 1.24	<0.0001*
Hematocrit	0.38 ± 0.00719	0.326 ± 0.01038	NS
Platelets (×10^6^/mL)	275 ± 12.00068	145 ± 9.57	<0.0001*
Birth weight (g)	3365 ± 165.39	2742 ± 197.97	<0.05
Gestational age (weeks) (at recruitment)	34.58 ± 0.48	34.11 ± 0.52	NS

Values represent mean ± SEM. Values compared between preeclamptic patients and normotensive patients, unpaired t‐test, *P < 0.05. SBP, systolic blood pressure; DBP, diastolic blood pressure; MAP, mean arterial pressure; BMI, body mass index; NS, not significant.

Microvascular blood flow was reduced in the preeclamptic group when compared with the normal pregnant controls (1.13 [0.94–1.54] vs. 4.06 [2.98–5.61] mL·min^−1^·100 mL^−1^, *P *<* *0.0001, respectively) and correlated inversely with systolic blood pressure, diastolic blood pressure, and MAP (*r* = −0.659, *P* < 0.0001; *r* = −0.586. *P* < 0.0001; *r* = −0.644, *P* < 0.0001) when including all pregnancies.

### Levels of inflammatory markers, antiangiogenic factors, and insulin resistance are elevated in preeclampsia

Serum PlGF was reduced in the preeclamptic group compared with normal pregnant women (26.5 [15.72–43.07] vs. 76.19 [62.95–182.56] pg/mL; *P *<* *0.005, respectively. Conversely, serum sFlt‐1 was increased in the preeclamptic group compared with the normotensive pregnancies (1448.69 [1173.29–1584.52] vs. 758.31 [639.11–1087.29] pg/mL, *P *<* *0.005, respectively) as was sEng (9.68 [9.49–10.02] vs. 4.44 [3.83–5.23] ng/mL; *P *<* *0.0001, respectively). All three biomarkers correlated with systolic blood pressure (*r* = −0.58, *P* < 0.005; *r* = 0.583 *P* < 0.005; *r* = 0.856 *P* < 0.0001, for PlGF, sFlt‐1, and sEng, respectively), diastolic blood pressure (*r* = −0.477, *P* < 0.01; *r* = 0.589 *P* < 0.005; *r* = 0.869 *P* < 0.0001, for PlGF, sFlt‐1, and sEng, respectively) and MAP (*r* = −0.644, *P* < 0.001; *r* = 0.591 *P* < 0.0001; *r* = 0.9 *P* < 0.0001, for PlGF, sFlt‐1, and sEng, respectively) when including all pregnancies.

Circulating markers of endothelial activation and inflammation (sICAM, sVCAM, e‐selectin, and TNF‐*α*) were elevated in the preeclamptic group when compared to normotensive controls (Table 2) as were circulating free glucose and insulin levels. When calculated as a homeostasis model of insulin resistance (HOMA‐IR), this was also significantly elevated in preeclamptic women when compared to normotensive controls (Table 2).

**Table 2:** Microvascular blood flow and circulating mediators

Variable	Normal pregnancy (*n* = 17)	Preeclampsia (*n* = 16)	*P* Value
Tissue blood flow (mL/100 mL/min)	4.06 (2.98–5.61)	1.13 (0.94–1.54)	<0.0001*
sICAM (ng/mL)	58.99 (44.43–71.80)	195.18 (184.9–239.3)	<0.0001*
sVCAM (ng/mL)	294.62 (239.48–331.26)	808.3 (703.37–866.11)	<0.0001*
eSelectin (ng/mL)	12.42 (4.23–22.09)^1^	24.35 (14.60–49.25)^2^	<0.05*
TNF‐*α* (pg/mL)	14.40 (12.92–15.57)	20.81 (19.23–22.71)	<0.0001*
PlGF (pg/mL)	76.19 (62.95–182.58)	26.54 (15.72–43.07)	<0.005*
sEng (ng/mL)	4.44 (3.83–5.23)	9.68 (9.49–10.02)	<0.0001*
sFlt‐1 (pg/mL)	758.31 (639.11–1087.29)	1448.69 (1173.29–1584.52)	<0.005*
sFlt‐1:PlGF (pg/mL)	9.14 (3.87–14.12)	45.16 (28.1–99.59)	<0.0001*
(sFlt‐1 + sEng):PlGF (pg/mL)	70.16 (27.19–111.22)	420.26 (250.21–728.67)	<0.0001*

Data represent median ± interquartile range. Unpaired t‐test between preeclamptic patients and normotensive patients, *P <* *0.05; ^1^(*n* = 16); ^2^(*n* = 15).

**Table 3:** Fasting glucose and insulin levels and insulin resistance (HOMA‐IR) at recruitment

Serum factor	Normal pregnancy (*n* = 17)	Preeclampsia (*n* = 16)	*P* Value
Free insulin (*μ*U/mL)	6.67 (5.42–10.21)	18.22 (16.98–21.56)	<0.0001*
Free glucose (mg/dL)	91.30 (83.49–103.0010)	121.32 (111.56–128.53)	<0.0001*
HOMA‐IR	1.69 (1.17–2.52)	5.50 (5.02–5.91)	<0.0001*

*P < 0.05.

### PlGF influences vascular function following multivariable adjustment

Analysis of both normotensive and preeclamptic subjects together with microvascular function set as the outcome measure demonstrated a significant relationship with PlGF (*r* = 0.878, *P* < 0.0001), that following adjustment for preeclampsia and circulating mediators that included: sFlt‐1, sEng, HOMA‐IR, TNF‐*α*, sICAM, sVCAM, and e‐selectin, was maintained as having the strongest relationship with vascular function (*β* = 0.825, *P* < 0.0001). The only other mediator to maintain a significant relationship with vascular function was sEng; however, the variance inflation factor was high enough (VF = 13.46) to suggest its behavior was nonspecific and influential in other areas.

### The correlation between (sFlt + sEng):PlGF and microvascular function is maintained in pregnancy independently of disease

Serum levels of PlGF, sEng, and sFlt‐1 were then expressed as a ratio of antiangiogenic:proangiogenic mediators that were elevated in preeclampsia when compared to normal pregnancy (420.26 [250.21–728.67] vs. 70.16 [27.19–111.22] pg/mL, *P *<* *0.0001, respectively).

**Figure 1:**
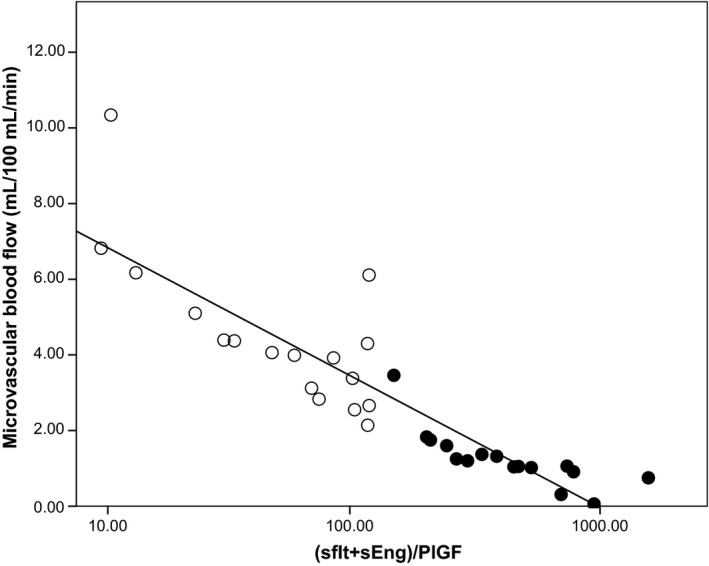
Relationship between (sFlt‐1 + sEng):PlGF (pg/mL) and microvascular blood flow. Open circles normotensive pregnancies, closed circles preeclamptic pregnancies. *R* ^2^ = −0.791.

The ratio of (sFlt+sEng):PlGF correlated with microvascular function when analyzed in all pregnancies (R^2^ = −0.791, *P* < 0.001), Figure 1. This ratio of (sFlt + sEng):PlGF also correlated with systolic blood pressure (*r* = 0.736, *P* < 0.0001), diastolic blood pressure (*r* = 0.614, *P* < 0.0001), and MAP (*r* = 0.695, *P* < 0.0001) across all pregnancies.

**Figure 2:**
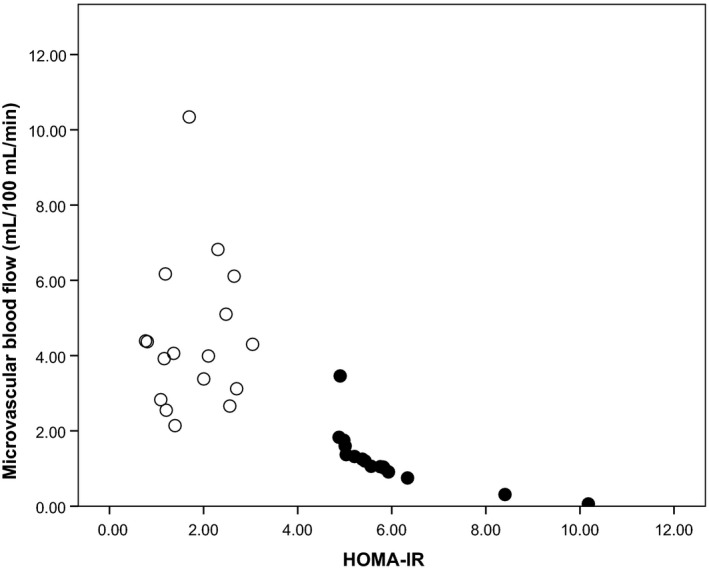
Relationship between insulin resistance (HOMA‐IR) and microvascular blood flow. Insulin resistance correlated with vascular function only upon analysis of the preeclamptic group and not in normotensive pregnancies (*r* = −0.991 *P* < 0.0001; *r* = 0.047, *P* = NS, respectively). Open circles normotensive pregnancies, closed circles preeclamptic pregnancies.

Insulin resistance correlated with vascular function upon analysis of the preeclamptic group but not in normotensive pregnancies (*r* = −0.991 *P* < 0.0001 and *r* = 0.047, *P* = NS, respectively), Figure 2.

**Figure 3:**
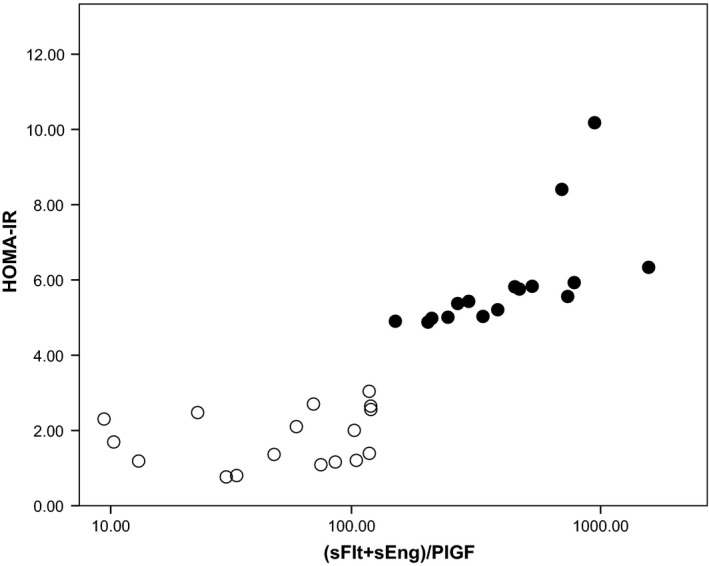
Relationship between insulin resistance (HOMA‐IR) and (sFlt‐1 + sEng):PlGF (pg/mL). Open circles normotensive pregnancies, closed circles preeclamptic pregnancies.

The suggestion of an effect modification or interaction of insulin resistance with the ratio of circulating angiogenic factors was explored further. Creating a new univariate model, the influence of the angiogenic factors combined with insulin resistance on microvascular function as an outcome measure was investigated using the interaction term ((sFlt + sEng):PlGF) x Insulin Resistance), and demonstrated statistical significance only in the preeclamptic cohort (*β* = 0.001, *P* < 0.05). This relationship clearly differentiated preeclamptic from normotensive pregnancies (Fig. 3).

## Discussion

This study is the first to examine the relationship between microvascular blood flow, circulating markers of angiogenesis, insulin resistance, and endothelial activation together in preeclampsia.

Decreases in microvascular blood flow were associated with elevations in antiangiogenic mediators in both healthy pregnancy and preeclampsia. The observed decrease in blood flow in preeclampsia was also associated with elevations in insulin resistance and circulating markers of endothelial activation and inflammation.

In pregnancy, transient increases in insulin resistance and inflammatory responses are considered normal alongside increased angiogenesis. Maternal venous capacitance increases, arterial resistance decreases, and vasodilatation increases are accompanied by an increase in blood volume (Carpenter 2007); however, in preeclampsia, these mechanisms become dysfunctional, manifested by maternal hypertension and end organ damage.

In the current study, we have found that the levels of circulating angiogenic biomarkers are strongly associated with vascular function independent of disease. The influence of angiogenesis on the progression of preeclampsia has been well established with the balance between pro‐ and antiangiogenic factors identifying women who will develop preeclampsia (Levine et al. 2004; Verlohren et al. 2012a; Chaiworapongsa et al. 2013). Whether these alterations are a primary event in the development of the disease or secondary to placental perfusion remains unclear. It is known that the preeclamptic maternal vasculature is dysfunctional, with recent evidence demonstrating a dysfunction in the production of mRNA and protein of antiangiogenic factors and proangiogenic factors in microvascular endothelial cells (Lee and Nevo 2016).

When the association of microvascular function and (sFlt + sEng):PlGF in preeclamptic women was coupled with insulin resistance, this relationship was amplified. It is known that the risk of preeclampsia associated with decreased PlGF levels are increased with hyperinsulinemia (Thadhani et al. 2004), suggesting that pathological alterations in either or both of these pathways may provide additive insults that create widespread injury.

This study, that includes the influence of sFlt, sEng, and PlGF with insulin resistance and microvascular dysfunction, highlights the importance of signaling pathways consequent to VEGF and insulin binding, suggest common pathways downstream of the receptors. Thus, alterations in signaling involving SFlt‐1, PlGF, and insulin may provide summative insults that lead to widespread endothelial activation and injury culminating in vascular dysfunction. Insulin itself has been considered a primary pathophysiological effector in hypertension (Wu et al. 1994), associated with elevated circulating noradrenaline (Anderson et al. 1991) and renal sodium retention (DeFronzo et al. 1976). Studies of the vasodilatory actions of insulin have revealed that insulin can act in a nitric oxide‐dependent fashion (Clark et al. 2003) that precedes the induction of glucose uptake (Vincent et al. 2004), suggestive of a primary effect of insulin on the vasculature and not secondary to changes in cellular metabolism.

In preeclampsia, the antiangiogenic imbalance of mediators that is associated with a decreased microvascular blood flow and exacerbated by high levels of insulin resistance are not observed in healthy pregnancy. These measurements could become a useful tool to assess the severity of multisystem dysfunction in preeclampsia as reduced tissue perfusion precedes end organ dysfunction.

Following pregnancy, it has been suggested that the persistence of subclinical inflammation, antiangiogenic proteins (Kvehaugen et al. 2011) and insulin resistance in women with preeclampsia results in an increased cardiovascular risk (Wolf et al. 2004) that potentially could be manifested as cardiovascular disease following exposure to other risk factors (Sattar and Greer 2002). Defining the risk associated with the ratio of antiangiogenic factors to proangiogenic factors, insulin resistance, and microvascular function justifies investigating this relationship earlier in pregnancy with an aim to promoting early intervention. Another strategy would be to investigate this disease after pregnancy to follow up any resolution of vascular damage, to promote early intervention and prevent future cardiovascular disease. Currently, no validated test exists that reliably predicts progression of preeclamptic pathology and therefore preeclamptic women who do not require immediate delivery are monitored as inpatients until timely delivery (Visintin et al. 2010). As the serum sFlt‐1:PIGF ratio correlates with the severity of preeclampsia (Verlohren et al. 2012b) and our data demonstrates that the microvascular blood flow (which precedes end organ dysfunction (Powe et al. 2011)) inversely correlates with both sFlt‐1:PIGF and sFlt‐1 +  sEng:PlGF, our data supports the clinical use of an antiangiogenic:proangiogenic ratio to identify preeclamptic women who may be at risk of underlying organ dysfunction and be more likely to deteriorate, requiring urgent intervention (Verlohren et al. 2012a) and early delivery.

### Limitations

A limitation of this study is the small sample size. Identifying interactions between variables is often difficult because of the requirement for large sample sizes for formal statistical testing; as a consequence these tests should only be used as a guide (Rothman and Greenland 1998). Although women in this study had mild preeclampsia, there was a significant relationship between microvascular blood flow, antiangiogenic factor ratio and insulin resistance.

We acknowledge that a subsequent study with a larger sample size including women with early onset and severe preeclampsia, and other conditions associated with insulin resistance would improve the accuracy of our findings in relation to the pro‐ and antiangiogenic balance.

## Conflict of Interest

The authors report no conflict of interest.
